# Anatomical and clinical variations in the mesoappendix and appendicular arteries: implications for appendicitis and surgical outcomes

**DOI:** 10.1111/ans.70090

**Published:** 2025-03-30

**Authors:** Mehmet Torun, Osman Sulak, Mukaddes Üçkarış, İsmail Ege Subaşı

**Affiliations:** ^1^ Gastrointestinal Surgery Clinic Erzurum State Hospital Erzurum Turkey; ^2^ Anatomy Departmant Üsküdar University Faculty of Medicine Istanbul Turkey; ^3^ Gastrointestinal Surgery Clinic University of Health Sciences Sancaktepe Dr.İlhan Varank Research and Training Hospital Istanbul Turkey

**Keywords:** anatomical variations, appendicitis, appendicular artery, mesoappendix, surgical complications, vascular anatomy

## Abstract

**Background:**

Anatomical variations in the mesoappendix and appendicular arteries play a crucial role in the development and management of appendicitis. This study aimed to analyze these variations and their clinical implications.

**Methods:**

A retrospective analysis of 287 patients who underwent appendectomy was conducted. Data on mesoappendix extension, appendicular arterial patterns, and appendicitis type were collected. Statistical analyses, including chi‐square tests and t‐tests, were performed to explore the relationships between variables.

**Results:**

The most common mesoappendix extension was ‘Whole Length’ (51.22%), followed by ‘Two‐thirds Length’ (32.06%). A ‘Single Artery’ supplied the appendix in 69.68% of cases, with the ileocolic artery being the primary origin (62.03%). Acute appendicitis is more common in younger patients, whereas chronic appendicitis is prevalent among older individuals. Significant associations were observed between mesoappendix extension and arterial origin (*P* < 0.001) as well as between age and appendicitis type (*P* = 0.034).

**Conclusion:**

This study highlights the anatomical and clinical variabilities of the mesoappendix and appendicular arteries. Understanding these variations is essential to optimize surgical outcomes and minimize complications. Future research should explore the implications of these findings in diverse populations.

## Graphical abstract

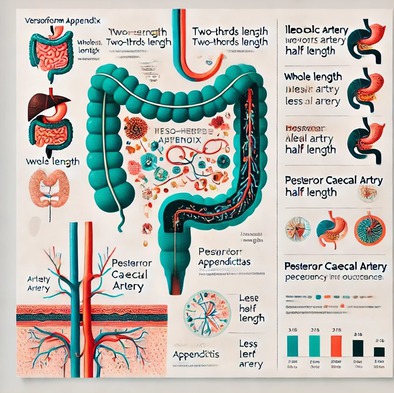

## Introduction

The vermiform appendix is a narrow, blind‐ended tube attached to the cecum and is historically considered a vestigial organ. However, recent studies suggest that the appendix plays an immunological role, particularly in maintaining gut flora homeostasis.^1^ Despite this potential function, it remains clinically significant, primarily because of its association with appendicitis, which is one of the most common surgical emergencies worldwide.^2^, ^3^ Understanding the anatomical variations of the appendix and its mesoappendix, which contains the appendicular artery, is crucial for evaluating the vascular supply, optimizing surgical techniques, and preventing complications during appendectomy.^4^

The mesoappendix is a peritoneal fold that connects the appendix to the ileum and cecum and encloses the appendicular artery. Its length and extent of attachment have significant clinical implications, as variations can influence the severity of appendicular inflammation, the risk of gangrene, and the potential spread of appendiceal tumours.^5^, ^6^ While some individuals have a ‘Whole Length’ mesoappendix extending to the tip of the appendix, others exhibit shorter extensions such as ‘Two‐thirds Length’ or ‘Half Length’, which may limit vascularization and increase susceptibility to ischemic complications.^7^

The appendicular artery, often a branch of the ileocolic artery, is typically an end artery, meaning that it has limited collateral circulation.^8^ This unique vascular supply makes the appendix particularly prone to ischemia in cases of thrombosis or severe inflammation.^9^ Additionally, studies have identified accessory appendicular arteries arising from various sources, including the posterior cecal artery and the arcade artery, which, if unrecognized, may lead to intraoperative bleeding and increased postoperative morbidity.^10^, ^11^ Recognizing these vascular variations is essential for preventing complications and ensuring safe and effective appendectomy procedures.^12^

Appendicitis, characterized by inflammation of the appendix, presents with variable clinical features depending on anatomical factors, such as appendix position, mesoappendix extension, and arterial supply. Acute appendicitis is more frequently observed in younger patients, whereas chronic appendicitis is more prevalent in older individuals.^13^, ^14^ Recent studies suggest that certain anatomical configurations, such as a short mesoappendix or aberrant arterial origins, may predispose patients to more severe appendicitis.^15^ Identifying these variations can improve diagnostic accuracy, guide surgical approaches, and enhance patient outcomes.^16^

Despite numerous studies on appendicular anatomy, there remains a gap in the understanding of how mesoappendix length and arterial variations correlate with appendicitis risk and surgical outcomes. This study aimed to provide a comprehensive analysis of mesoappendix extension, appendicular arterial patterns, and their clinical implications. By integrating anatomical, pathological, and clinical perspectives, we hope to contribute valuable insights to the existing literature and provide a foundation for improved surgical decision‐making and patient care.^17^, ^18^

## Material and methods

### Study design

This study was designed as a retrospective observational analysis of the anatomical and clinical data related to appendicular vascular patterns, mesoappendix extension, and appendicitis classification. Data were collected from 287 patients who had undergone appendectomy between September 2023 and September 2024.

### Study population

This study included patients with a confirmed diagnosis of appendicitis based on clinical, radiological, and pathological findings. Patients were excluded if they had incomplete data, prior appendicular surgery, or congenital anomalies involving the appendix. The final cohort consisted of 287 patients, aged 18–80 years.

### Ethical considerations

This study was conducted in compliance with the principles of the Declaration of Helsinki. Ethical approval was not required, as this study involved a retrospective analysis of anonymized data without direct patient interaction. Institutional approval was obtained for the data access and use.

### Data collection

Patient records, including operative notes and histopathology reports, were reviewed to extract relevant data. Key variables included mesoappendix extension, categorized as ‘Whole Length’, ‘Two‐thirds Length’, ‘Half Length’, or ‘Less than Half Length’, along with arterial type, classified as ‘Single Artery’, ‘Double Artery’, or ‘Accessory Artery’. The origin of the appendicular artery was determined intraoperatively and documented as arising from the ileocolic, ileal, posterior cecal, anterior cecal, or arcade arteries. The appendicitis type was classified as ‘Acute’ or ‘Chronic’ based on histopathological findings. Demographic data, including patient age, sex, and relevant clinical history, were collected to assess potential correlations between anatomical variations and appendicitis presentation.

### Statistical analysis

Statistical analysis was conducted using specialized software to ensure accurate evaluation of the collected data. Descriptive statistics were applied to summarize categorical variables as frequencies and percentages, whereas continuous variables were expressed as means with standard deviations. Various statistical tests were used to assess the associations and differences between the key study parameters. Chi‐square tests were performed to evaluate the relationships between categorical variables, including mesoappendix extension and arterial type or origin. Independent *t*‐tests were used to compare the mean age differences between the patients with acute and chronic appendicitis. Additionally, a cross‐tabulation analysis was conducted to examine the distribution of appendicitis types across different age groups. A significance level of *P* < 0.05 was used to determine statistical significance across all analyses, ensuring robust and reliable findings.

## Results

The current study analyzed the data of 287 patients to evaluate the anatomical and clinical characteristics associated with mesoappendix extension, appendicular arterial patterns, and appendicitis type.

**Fig. 1:**
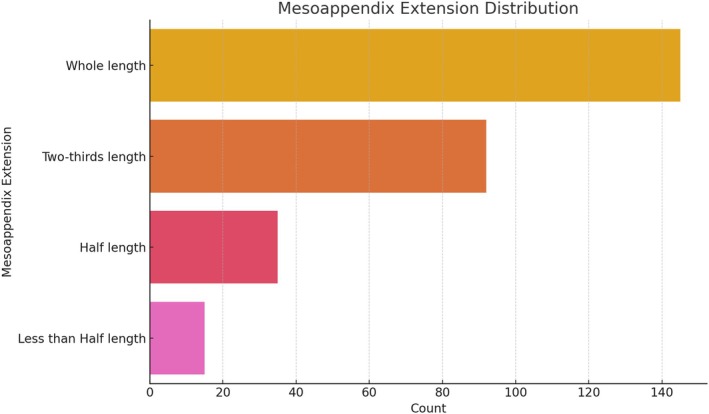
Distribution of mesoappendix extension.

**Fig. 2:**
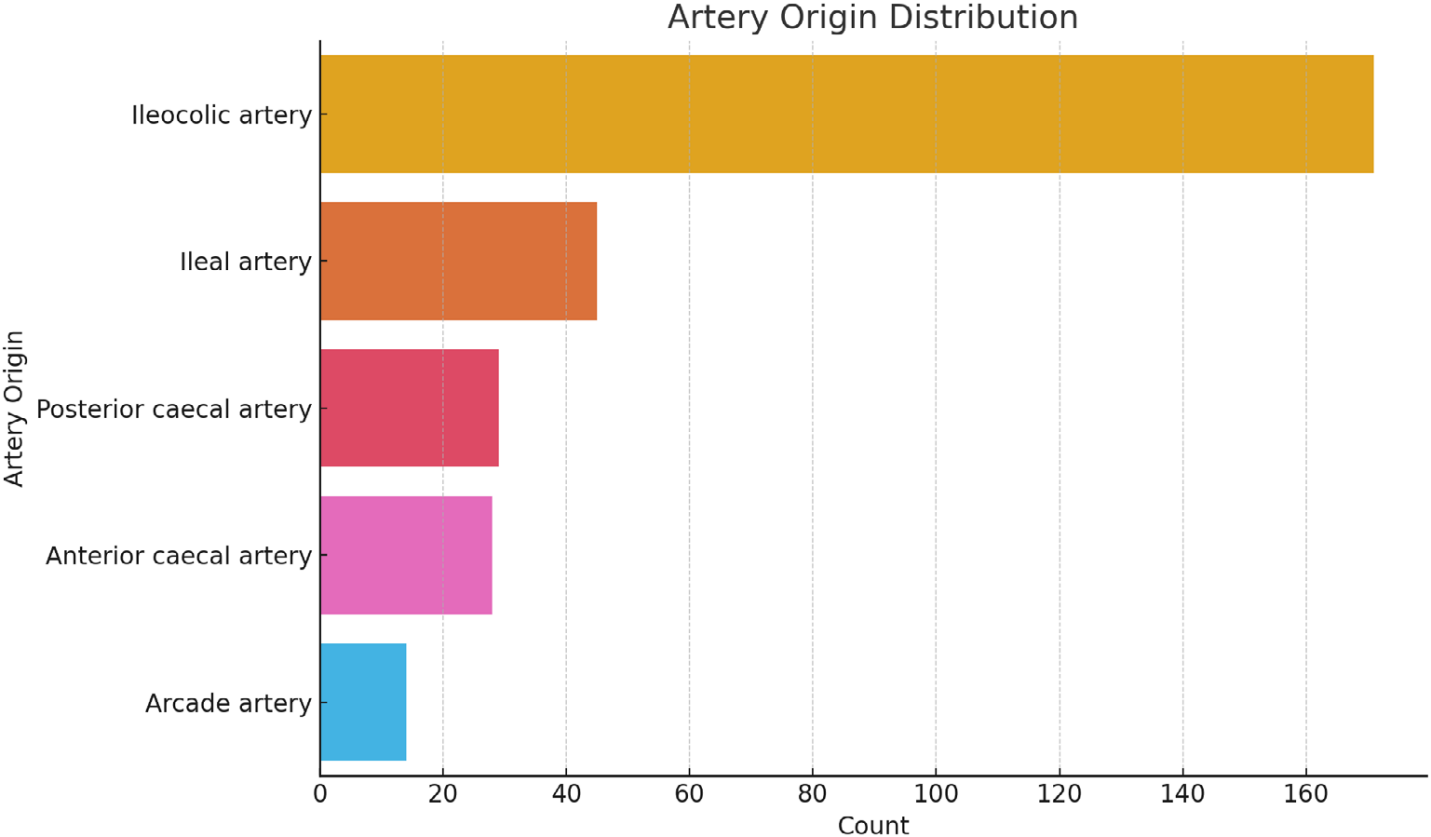
Variations in the origin of the appendicular artery.

**Fig. 3:**
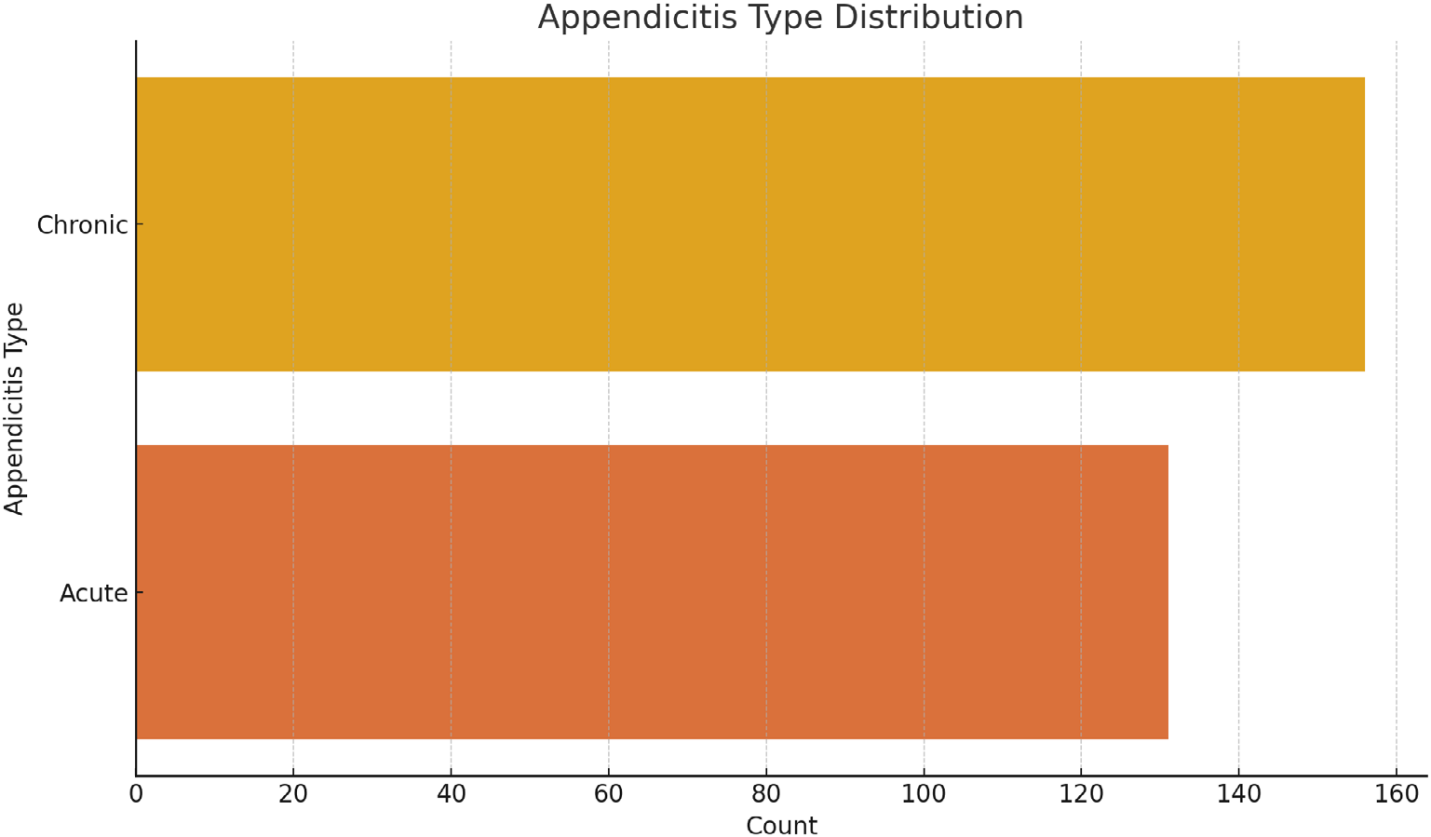
Relationship between mesoappendix extension and appendicitis type.

**Fig. 4:**
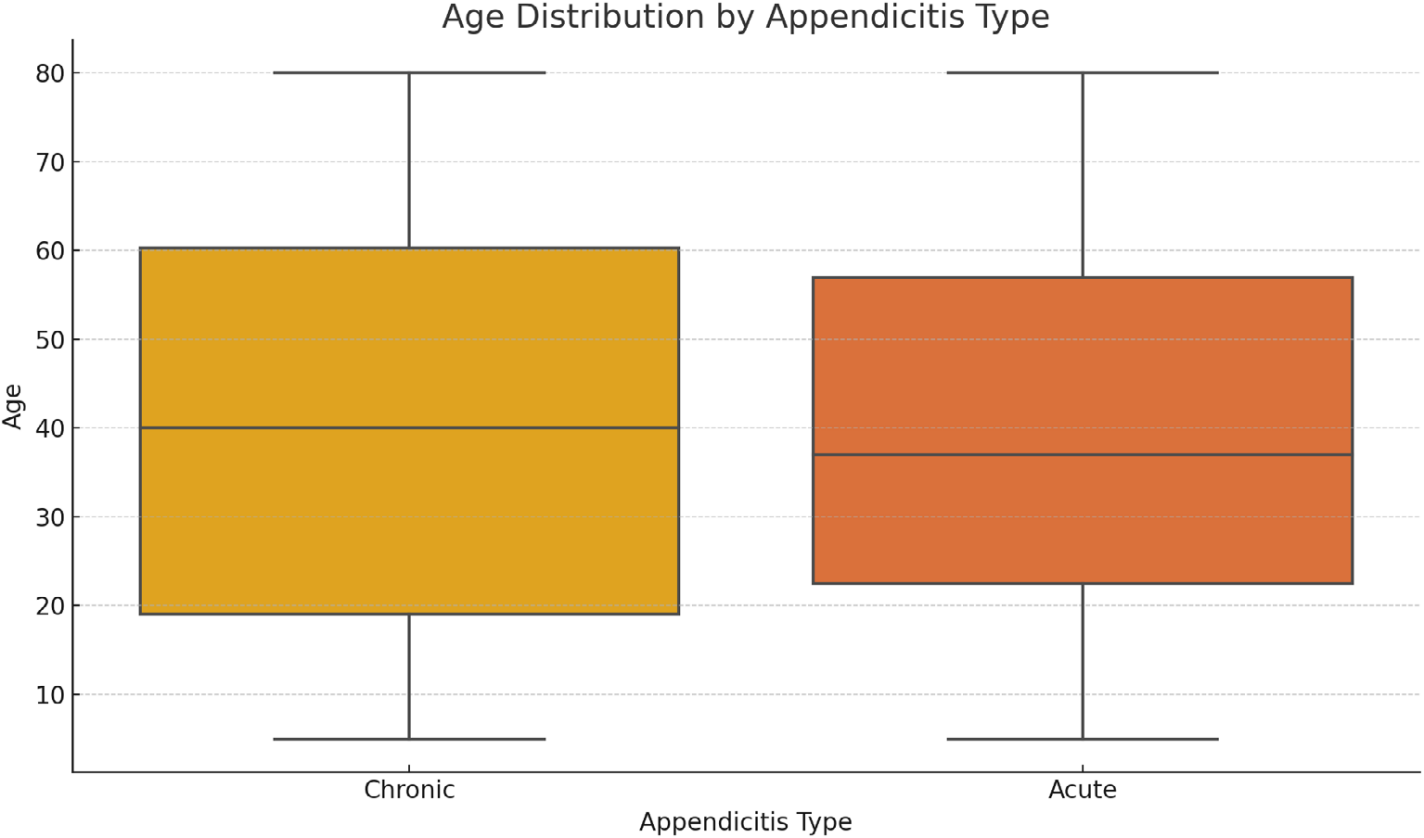
Comparison of age distribution in acute and chronic appendicitis.

The anatomical and clinical distributions of mesoappendix and appendicular artery variations are presented in Table 1. The majority of patients (51.22%) demonstrated a ‘Whole Length’ mesoappendix extension, while ‘Two‐thirds Length’ was observed in 32.06%, ‘Half Length’ in 12.54%, and ‘Less than Half Length’ in 4.18% of the cases. This distribution emphasizes the variability in mesoappendix length among the studied populations (Fig. 1). The distribution of arterial types showed that 69.68% of patients were supplied by a ‘Single Artery’, while 20.24% had a ‘Double Artery’, and 9.76% had an ‘Accessory Artery’. The primary origins of the main appendicular artery were the ileocolic artery (62.03%), ileal artery (15.88%), posterior cecal artery (9.76%), anterior cecal artery (7.45%), and arcade artery (4.88%) (Fig. 2). Of the 287 cases, 54.36% were classified as ‘Chronic Appendicitis’, whereas 45.64% were ‘Acute Appendicitis’ (Fig. 3). The age of patients ranged from 18 to 80 years, with a mean age of 47.2 years (SD ± 15.3). Males constituted 53.5% of the population, and females accounted for 46.5% of the population (Fig. 4).

**Table 1:** Anatomical and clinical distribution of mesoappendix and appendicular artery variations

Variable	Category	Frequency (*n*)	Percentage (%)
Mesoappendix extension	Whole length	147	51.22
	Two‐thirds length	92	32.06
	Half length	36	12.54
	Less than half length	12	4.18
Appendicular artery type	Single artery	200	69.68
	Double artery	58	20.24
	Accessory artery	28	9.76
Appendicular artery origin	Ileocolic artery	178	62.03
	Ileal artery	46	15.88
	Posterior caecal artery	28	9.76
	Anterior caecal artery	21	7.45
	Arcade artery	14	4.88
Appendicitis type	Chronic appendicitis	156	54.36
	Acute appendicitis	131	45.64

Statistical tests were used to identify significant relationships between the categorical and continuous variables (Table 2).

**Table 2:** Statistical analysis of relationships between variables

Variable comparison	Statistical test	Test statistic	*P*‐value
Mesoappendix extension & artery type	Chi‐square	*χ* ^2^ = 2.89	0.235
Mesoappendix extension & arterial origin	Chi‐square	*χ* ^2^ = 18.45	<0.001
Artery type & appendicitis type	Chi‐square	*χ* ^2^ = 1.56	0.457
Age & appendicitis type	*t*‐test	*t* = 2.13	0.034
Gender & appendicitis type	Chi‐square	*χ* ^2^ = 0.82	0.664
Mesoappendix extension & appendicitis type	Chi‐square	*χ* ^2^ = 2.89	0.235
Age groups & appendicitis type	Chi‐square	*χ* ^2^ = 4.34	0.227

A chi‐square test revealed no statistically significant association between mesoappendix extension and artery type (*χ* ^2^ = 2.89, *P* = 0.235), indicating that the type of appendicular artery was independent of mesoappendix length. A significant relationship was observed between mesoappendix extension and arterial origin (*χ* ^2^ = 18.45; *P* < 0.001). Patients with ‘Whole Length’ mesoappendix were more likely to have an arterial supply originating from the ileocolic artery. There was no significant association between artery type and appendicitis (*χ* ^2^ = 1.56, *P* = 0.457). A *t*‐test comparing the mean age of the patients with acute versus chronic appendicitis showed a significant difference (*t* = 2.13, *P* = 0.034). Patients with chronic appendicitis were older, on average, than those with acute appendicitis.

To assess potential sex differences, we performed a chi‐square test to evaluate the relationship between sex and appendicitis type. The analysis revealed no statistically significant association between the two variables (*χ* ^2^ = 0.82, *P* = 0.664). Additionally, there was no significant difference in mesoappendix extension or arterial supply patterns between male and female patients.

A chi‐squared test was conducted to evaluate the relationship between mesoappendix extension and appendicitis type. The analysis revealed no statistically significant association between the two variables (*χ* ^2^ = 2.89, *P* = 0.235).

Cross‐tabulation of age groups and appendicitis type revealed that acute appendicitis was more prevalent among younger patients (0–18 years: 70%), whereas chronic appendicitis was more common in older age groups (61–80 years: 65%). However, the chi‐squared test for this relationship was not statistically significant (*χ* ^2^ = 4.34, *P* = 0.227).

## Discussion

Anatomical and clinical variations in the appendix, including mesoappendix extension and appendicular arterial patterns, are crucial considerations for understanding appendicitis and optimizing surgical management. This study adds to the growing body of literature on these variations by providing detailed anatomical and clinical correlations and offering valuable insights for surgeons.

### Mesoappendix extension and surgical implications

The mesoappendix plays a vital role in vascularization of the vermiform appendix. Our findings revealed that a ‘Whole Length’ mesoappendix extension was the most prevalent type (51.22%), aligning with previous studies that reported similar distributions.^19^, ^20^ A ‘Two‐thirds Length’ extension was observed in 32.06% of cases, consistent with findings from studies conducted in different populations, including reports from India and Europe.^6^ The presence of shorter mesoappendix extensions, such as ‘Half Length’ or ‘Less than Half Length’, poses a higher risk for ischemia and gangrene due to limited vascular support.^8^ These findings emphasize the importance of recognizing mesoappendix variations during appendectomy in order to reduce surgical complications.

### Appendicular artery variability and clinical relevance

The appendicular artery, often described as the end artery, exhibited considerable variability in this study. A ‘Single Artery’ was the most common arterial type (69.68%), consistent with the results of prior cadaveric studies.^11^ However, the presence of ‘Accessory Arteries’ (9.76%) warrants attention, as these vessels can lead to intraoperative haemorrhage if not adequately ligated.^9^ The primary appendicular artery originated from the ileocolic artery in 62.03% of cases, which aligns with earlier reports of appendicular vascularization.^10^ Less frequently observed arterial origins, such as the ileal, posterior cecal, and arcade arteries, highlight the complexity of appendicular blood supply.^12^ From a surgical perspective, understanding these vascular variations is crucial for preventing unexpected bleeding and ensuring complete ligation during appendectomy.^18^

### Appendicitis type and demographic trends

Clinical implications of appendicular vascular variations are evident in appendicitis. Acute appendicitis was observed in 45.64% of the cases, while chronic appendicitis accounted for 54.36%. This distribution reflects the demographic and pathological trends observed in previous epidemiological studies.^2^, ^4^ Younger patients are more likely to present with acute appendicitis, whereas chronic appendicitis is more prevalent among older individuals, supporting the findings of previous reports suggesting that delayed diagnosis or prolonged subclinical inflammation may contribute to chronic appendicitis.^13^

Interestingly, no significant association was found between mesoappendix extension and appendicitis type (*P* = 0.235), suggesting that the anatomical length of the mesoappendix may not directly influence the inflammatory process. However, a strong correlation was observed between mesoappendix extension and arterial origin (*P* < 0.001), with patients having a ‘Whole Length’ mesoappendix more likely to receive arterial supply from the ileocolic artery. This finding agrees with anatomical studies indicating that mesoappendix length influences vascular branching patterns.^5^

The lack of a significant association between sex and appendicitis type (*P* = 0.664) contradicts previous studies that suggested male predominance in acute appendicitis.^14^ This discrepancy may be attributed to regional or demographic differences in the study population. In contrast, the observed significant age‐related differences in appendicitis types (*P* = 0.034) emphasize the need for age‐specific diagnostic and management strategies.^7^

### Surgical considerations and future directions

From a surgical standpoint, it is essential to recognize the anatomical variability of the mesoappendix and appendicular arteries. The presence of accessory arteries or aberrant arterial origins, such as the arcade artery, requires careful dissection to avoid intraoperative bleeding.^7^, ^17^ Additionally, surgeons should consider the implications of a short mesoappendix, which can increase the risk of ischemia and necessitate prompt intervention.^16^

This study has certain limitations, including its retrospective design and reliance on intraoperative observations, which may have introduced observer bias. Future research could benefit from advanced imaging techniques such as angiography to provide more precise documentation of appendicular vascular patterns.^21^ Additionally, larger multicenter studies could validate these findings and explore their implications across diverse populations.^3^

In conclusion, this study provides valuable insights into anatomical and clinical variations of the appendix. These findings highlight the importance of understanding mesoappendix extension and arterial patterns to enhance surgical outcomes and minimize complications. Further research is needed to explore the interplay between these anatomical variations and appendicitis progression, thus paving the way for more tailored surgical approaches.

## Author contributions

**Mehmet Torun:** Conceptualization; investigation; writing – original draft; methodology; validation; visualization; writing – review & editing; software; formal analysis; project administration; data curation; resources. **Osman Sulak:** Writing – original draft; methodology; validation; formal analysis; supervision; data curation. **Mukaddes Üçkarış:** Conceptualization; methodology; validation; software; formal analysis; project administration; data curation. **İsmail Ege Subaşı:** Conceptualization; investigation; writing – original draft; methodology; validation; visualization; writing – review & editing; software; formal analysis; project administration; data curation; supervision; resources. All authors have read and approved the final manuscript and agree to be accountable for all aspects of the work in ensuring its integrity and accuracy.

## Conflict of Interest Statement

The authors declare that they have no conflicts of interest related to this study. There are no financial, personal, or professional relationships that could be perceived as influencing the findings or interpretations presented in this manuscript.

## References

[1] Laurin M , Everett ML , Parker W . The cecal appendix: one more immune component with a function disturbed by post‐industrial culture. Anat. Rec. (Hoboken). 2011; 294: 567–579. 21370495 10.1002/ar.21357

[2] Addiss DG , Shaffer N , Fowler BS , Tauxe RV . The epidemiology of appendicitis and appendectomy in the United States. Am. J. Epidemiol. 1990; 132: 910–925. 2239906 10.1093/oxfordjournals.aje.a115734

[3] Schwartz SJ , Shires GT , Spencer FC *et al*. Principles of Surgery, 7th edn. McGraw‐Hill, 1999.

[4] Anderson RE , Houganer A , Thalin AJD . Diagnostic accuracy and perforation rate in appendicitis: association with age and sex of patients. Eur. J. Surg. 1992; 158: 37–41. 1348639

[5] Buschard K , Kjaeldgaard A . Investigation and analysis of the position, length, and embryology of the vermiform appendix. Acta Chir. Scand. 1973; 139: 293–298. 4698491

[6] Geethanjali HT , Subhash LP . A study of variations in the position of vermiform appendix. Anat. Karnataka 2011; 5: 17–23.

[7] Iqbal T , Amanullah A , Nawaz R . Pattern and positions of the vermiform appendix in people of Bannu district. Gomal J. Med. Sci. 2012; 10: 100–103.

[8] Ajmani ML , Ajmani K . The position, length, and arterial supply of the vermiform appendix. Anat. Anz. 1983; 153: 369–374. 6881534

[9] Rahman MM , Khalil M , Sultana SZ *et al*. Extent of mesoappendix in Bangladeshi people. J. Bangladesh Soc. Physiol. 2009; 4: 20–23.

[10] Ouattara D , Kipré YZ , Broalet E *et al*. Classification of the terminal arterial vascularization of the appendix. Surg. Radiol. Anat. 2007; 29: 635–641. 17968483 10.1007/s00276-007-0265-6

[11] Veeresh H . A study of arterial supply of vermiform appendix in humans. J. Evol. Med. Dent. Sci. 2012; 1: 807.

[12] Solanke TF . The blood supply of vermiform appendix in Nigerians. J. Anat. 1968; 102: 353–361. 5643847 PMC1231322

[13] Bakheit MA , Warille AA . Anomalies of the vermiform appendix and prevalence of acute appendicitis in Khartoum. East Afr. Med. J. 1999; 16: 338–340. 10750522

[14] Lohar HP , Calcuttawala MA , Nirhale DS *et al*. Epidemiological aspects of appendicitis in a rural setup. Med. J. DY Patil Univ. 2014; 7: 753–757.

[15] Nirmaladevi M , Seshayyan S . Cadaveric study on the origin of the appendicular artery. Int. J. Anat. Res. 2016; 4: 1769–1771.

[16] Seshachalam T , Gorur SR . The arterial supply of the appendix. Indian Med. Gaz. 1930; 65: 693–694. PMC5157511 29008881

[17] Mitroffanof P . Transplantation of the appendix for incontinence bladder treatment. Surg. Radiol. Anat. 1980; 29: 635–641.

[18] Nuchhi AB , Yatagiri SV , Patil BG , Bannur BM . Study of arterial supply of caecum and appendix: a cadaveric study. Int. J. Anat. Res. 2017; 5: 4158–4162.

[19] Bush CM , Campbell JR , Pemberton JH . The mesoappendix: an anatomic and clinical review. Clin. Anat. 2012; 25: 302–307.

[20] Loukas M , Stewart D , Louis RG *et al*. The vascular supply of the vermiform appendix: a review. Surg. Radiol. Anat. 2005; 27: 399–405.

[21] Sabiston DC , Townsend CM , Beauchamp RD . Sabiston Textbook of Surgery, 16th edn. W.B. Saunders, 2001.

